# Core-shell nanoparticle arrays double the strength of steel

**DOI:** 10.1038/srep42547

**Published:** 2017-02-22

**Authors:** J.-B. Seol, S.-H. Na, B. Gault, J.-E. Kim, J.-C. Han, C.-G. Park, D. Raabe

**Affiliations:** 1 National Institute for Nanomaterials Technology, POSTECH, Pohang 37673, South Korea; 2 Department of Materials Science and Engineering, POSTECH, Pohang 37673, South Korea; 3 Max-Planck-Institut für Eisenforschung, Max-Planck-Straβe 1, D-40237 Düsseldorf, Germany

## Abstract

Manipulating structure, defects and composition of a material at the atomic scale for enhancing its physical or mechanical properties is referred to as nanostructuring. Here, by combining advanced microscopy techniques, we unveil how formation of highly regular nano-arrays of nanoparticles doubles the strength of an Fe-based alloy, doped with Ti, Mo, and V, from 500 MPa to 1 GPa, upon prolonged heat treatment. The nanoparticles form at moving heterophase interfaces during cooling from the high-temperature face-centered cubic austenite to the body-centered cubic ferrite phase. We observe MoC and TiC nanoparticles at early precipitation stages as well as core-shell nanoparticles with a Ti-C rich core and a Mo-V rich shell at later precipitation stages. The core-shell structure hampers particle coarsening, enhancing the material’s strength. Designing such highly organized metallic core-shell nanoparticle arrays provides a new pathway for developing a wide range of stable nano-architectured engineering metallic alloys with drastically enhanced properties.

Very strong materials often lack ductility, and ductile materials are usually not strong; this is known as the strength-ductility trade-off^1^. Mechanical properties of materials are directly linked to their internal structural defects, and how easily they can move. Amongst those defects, dislocations that are linear irregularities in the crystal lattice, are the primary carriers of shear, enabling the well-known formability of metallic materials^2^.

Nanostructuring is a successful strategy to manipulate the internal defect landscape of metallic alloys for improving their strength while maintaining ductility^1^,^2^,^3^,^4^,^5^,^6^. This strategy has been widely used to produce nanocystalline metallic materials, in which a high number density of grain boundaries (GBs) impedes lattice dislocation motion, thereby providing strength to materials used in manufacturing, construction, energy supply and transportation^7^,^8^,^9^,^10^. However, nanocystalline alloys are metastable, and the driving force for grain growth at moderate temperatures can be sufficient to trigger a drastic modification of their fine-scale substructure and a loss of the material’s properties of interest. Also, most nanomaterials cannot be readily synthesized at large quantities.

An alternative approach is to exploit the formation of nanometer-sized precipitates through a simple heat treatment. These precipitates act as nanoparticles (NPs) dispersed in a solid metallic matrix and provide effective obstacles for dislocation motion, thereby increasing yield strength, high-temperature strength, and creep resistance of numerous engineering alloys^11^,^12^,^13^,^14^. In body-centered cubic (bcc) alloys, strengthening via dispersion of an ultrahigh number density of NPs can be achieved by adding small quantities (≤2 wt.%) of elements (e.g. Ti, Nb, Mo, Ta, V) that promote their formation. When finely dispersed, these NPs leave only narrow spaces for dislocations to move freely, so dislocations must curve around or cut through NPs to sweep the material during deformation. This process requires relatively large amounts of energy, so that such NP dispersion increases the flow stress substantially, a mechanism known as Orowan effect^13^,^14^.

This effect can be even substantially enhanced by using regular arrangements of NPs instead of a random dispersion, where ‘regular’ refers to linear and nearly equally spaced NP alignments along <110> while in ‘random’ arrays NPs have no such order. The substantially larger interparticle spacings occurring in random obstacle fields leave more room for dislocations to bow out and shear the material at lower stresses, as compared to regular NP arrays which more efficiently hinder dislocation motion^13^,^14^.

A possible route towards a substructure architecture containing a regular and very dense array of NPs in bcc Fe-based alloys is interphase precipitation^15^,^16^,^17^,^18^,^19^,^20^,^21^,^22^,^23^,^24^,^25^. This is a process whereby bulk phase transformation and NP formation occur during the same heat treatment. This can be achieved by adjusting chemical composition, temperature and kinetics^21^,^22^,^23^,^24^,^25^. In the present case we realized this concept as follows: During cooling from high-temperatures of ~1200 °C, the face-centered cubic (fcc) high temperature phase transforms to the low-temperature bcc product phase. During transformation, NPs permanently nucleate on and detach from the moving heterophase interfaces that enable growth of the low temperature bcc phase at the expense of the fcc high temperature phase^18^,^19^,^20^,^21^. After cooling, a large density of interphase-precipitated NPs has formed with a diameter below 20 nm and an average interparticle spacing below 15 nm. Permanent nucleation and detachment of the NPs on the moving interfaces lead to their linear alignment in the matrix^15^,^18^,^19^,^20^,^21^,^22^,^23^,^24^,^25^. This non-random NP distribution can increase the tensile strength of Ti–Mo–bearing bcc alloys^15^,^18^ upon heat treatment at 600 °C for 60 min up to values of 995 MPa^15^ and the yield strength (at 0.2% offset) up to 945 MPa^15^. The main challenge associated with these NPs though is that they are not intrinsically stable, i.e. they are susceptible to capillarity–driven competitive growth, referred to as Ostwald ripening. This effect may substantially reduce their strengthening capacity over time and upon exposure to elevated temperatures^26^.

Here we report on a novel nanostructuring approach that combines the transformation and interphase precipitation phenomena outlined above with a topologically highly organized, ultrahigh number density of thermally stable core-shell NPs formed during heat treatment at 640 °C for up to 360 min. The concept is applied to a plain bcc Fe alloy, doped with Ti, Mo, and V, doubling its ultimate tensile strength from 500 MPa to 1 GPa. For better clarity, the tensile strength and yield strength of the present alloys were compared with those of the aforementioned alloys, doped with Ti and Mo, subject to shorter heat treatment times (below 60 min) (see Supplementary Table S1).

By using atom probe tomography (APT) and high-resolution transmission electron microscopy (HRTEM), we explore the interface-stabilized core-shell NPs and their strictly regular arrangement. We use an Fe–2.0 Mn–0.2 Si–0.1 Al–0.2C model alloy containing a total of 0.3 at. % Ti, Mo, and V. This alloy was subject to isothermal heat treatments at 640 °C for 30, 60, or 360 min, hereafter referred to as samples NP-30, NP-60, and NP-360, respectively (see Methods and Supplementary Fig. S1).

## Results

### Tensile property measurement

**Figure 1:**
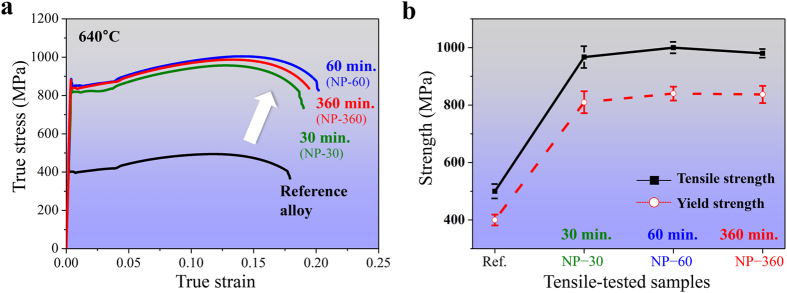
Uniaxial tensile true stress-true strain curves of an Fe-based alloy, doped with Ti, Mo, and V, and of an un-doped reference sample. (**a**) Tensile test data for heat-treated samples containing the nanoparticle-forming elements Ti, Mo and V and for a heat treated reference alloy without particle-forming elements. (**b**) Tensile strength (blank rectangles) and yield strength (open circles) taken for three samples at each heat treatment time. For comparison, tensile strength and yield strength of the reference alloy are included. The material is an Fe–2.0 Mn–0.2 Si–0.1 Al–0.2C alloy containing a total of 0.3 at. % Ti, Mo, and V. Samples subjected to heat treatments at 640 °C for 30, 60, or 360 min are referred to as NP-30, NP-60, and NP-360, respectively.

Uniaxial tensile test data obtained from the heat treated samples containing Ti, Mo, and V are compared to a Ti-Mo-V-free reference material, also annealed at 640 °C for 60 min. The tensile true stress−true strain curves demonstrate the enormous effect of the Ti–Mo–V addition on the mechanical behavior of the alloy (Fig. 1). The mechanical properties of all the samples were characterized by means of ultimate tensile strength (UTS) and 0.2% offset yield strength (YS). Two distinct features are observed. First, the material’s strength is doubled when doping Ti, Mo, and V, from a tensile strength of 500 MPa for the non-doped reference material to 1 GPa for the doped alloy. On an average, the YS reached 830 MPa from 400 MPa upon doping with Ti, Mo, and V. Second, the material fully maintains its ductility even though its strength is twice as high as before, i.e. elongation to fracture for all samples is 18–21% regardless of the heat treatment times.

### Microstructure characterization

**Figure 2:**
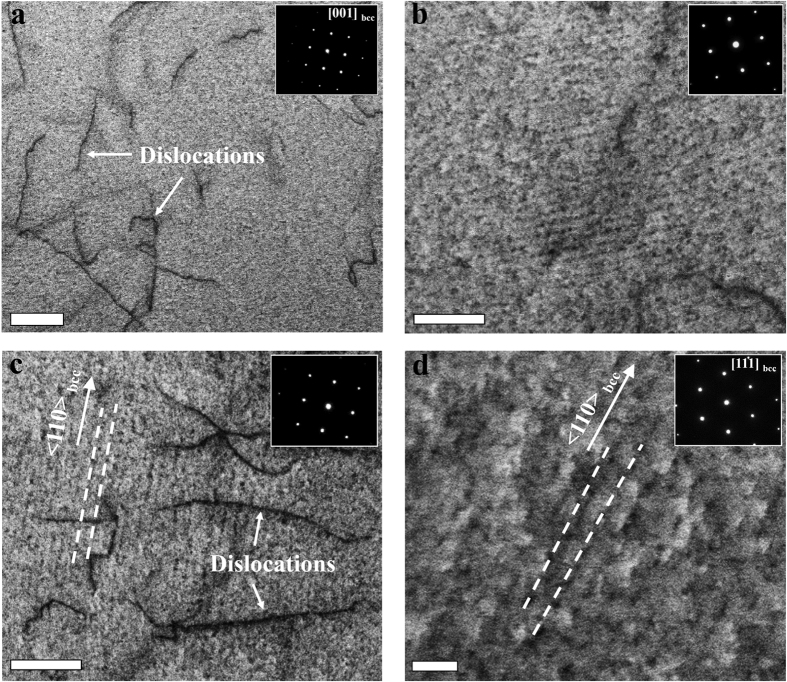
Microstructure of an un-doped reference sample and an Fe-based alloy, doped with Ti, Mo, and V, showing dislocations and an ultrahigh number density of nanoparticles with non-random distribution. Bright-field TEM images and the corresponding selected area electron diffraction patterns with a [001]_bcc_ zone axis shown as inset for (**a**) the reference alloys showing an interphase-precipitated nanoparticle-free matrix, and (**b**) sample NP-30 (640 °C 30 min.) showing the highly dense NPs with a non-random dispersion. (**c,d**) Bright-field TEM images of sample NP-60 (640 °C 60 min.) showing a chain-like distribution of the nanoparticles within a nearly-uniform nanoparticle array: (**c**) with the diffraction pattern indexed as [001]_bcc_; (**d**) different bcc grain observed from a different diffraction pattern with a [111]_bcc_ zone axis shown as inset, revealing planar nanoparticle arrays parallel to the <110> crystal direction, indicated by the dashed lines; scale bar, 40 nm. Scale bars for (**a–c**) are 100 nm.

To better understand these phenomena, we performed TEM on the reference material and on the NP-30 samples in crystals aligned for probing the [001]_bcc_ zone axis^21^,^22^. As expected, the non-doped reference sample does not show any NPs in the bright-field TEM micrographs or diffraction patterns (Fig. 2a). In contrast, the Ti–Mo–V doped NP-30 sample contains a highly structured nano-array of NPs with an average size <5 nm (Fig. 2b). The NPs were characterized in high detail by correlative TEM and APT analysis, focusing here on the NP-60 sample (Fig. 2c,d). The dislocations and NPs produce darker contrast than the bcc matrix. No NP reflection spots appear in the [001]_bcc_ selected area electron diffraction pattern (inset), because of their small volume fraction relative to the adjacent bcc matrix. The average size of the NPs in this sample is below 15 nm. Imaging the NPs in diffraction conditions along a different zone axis, i.e. [111]_bcc_ (Fig. 2d), confirms their surprisingly regular arrangement, i.e. they form highly organized planar nano-arrays consisting of parallel sets of linearly aligned NP chains. These NP chains are aligned in the <110> direction of the bcc host crystal, as indicated by the dashed lines superimposed within the TEM image. The average spacing among the linear NP chains is 9–12 nm.

**Figure 3:**
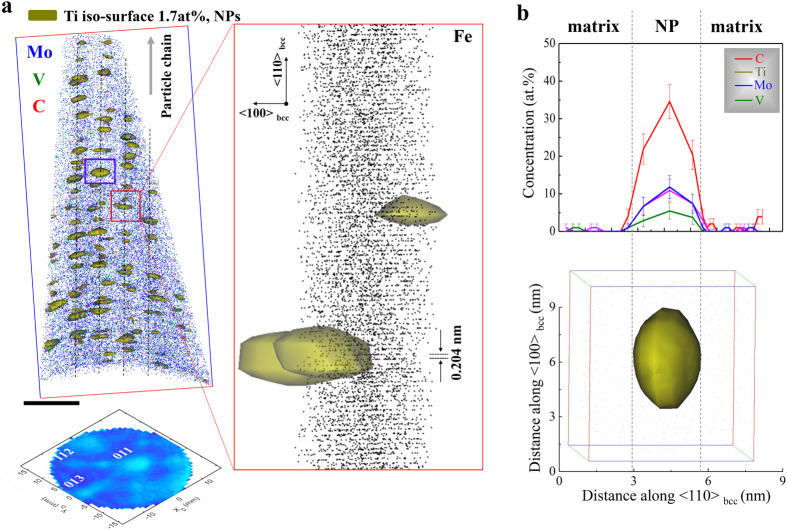
APT analysis of sample NP-60 (640 °C 60 min). (**a**) 3D atom map of Mo, V and C in the APT reconstruction with a volume of 41 × 41 × 85 nm^3^, using 1.7 atomic % Ti isoconcentration envelopes (1.7 atomic % of Ti was chosen as a threshold value to display the nanoparticles) and the corresponding detector desorption map (lower left inset) that was tilted to allow for direct comparison with the reconstructed image; scale bar, 20 nm. Blue: Mo, green: V, and red: C atoms. In the detector map, the color scales with the local hit-density on the APT detector (dark blue: high-density, sky blue: low-density). Nearly-uniform arrangement of nanoparticles assembled in the array. An expanded view of the individual nanoparticles in the red box shows the {110} atomic planes of a bcc host phase (Fe, black dots) with the measured interplanar spacing, revealing the growth of nanoparticles parallel to the <100> bcc phase. (**b**) Detailed analysis of a representative NP marked by the purple box in (**a**); the different projection and compositional profiles of Ti, Mo, V and C across the interfaces between the matrix and the NP. Errors bars given in (**b**) represent the 2*σ* measurements.

Since interparticle spacing data obtained from TEM imaging may suffer from projection effects^21^,^24^, we also employed APT analysis. We image a set of NPs highlighted in terms of 1.7 at. % Ti isoconcentration envelops in the NP-60 sample in Fig. 3. The average number density of the NPs is approx. 6.8 × 10^23^ m^−3^, which is nearly constant regardless of the applied heat treatment times (see Supplementary Table S2). For comparison, TEM results in conjunction with carbon replica methods are included. Note that the TEM-measured nanoparticle number densities were lower than the APT-measured ones, owing to the limitation of carbon replica methods with respect to nanoparticles with very small size. The average interparticle spacing within each of the planar arrays is in the range of 3–10 nm. Figure 3a shows that atom probe crystallography^27^ reveals the {011} atomic planes of the bcc host phase with an interplanar spacing of 0.204 nm together some NPs. We note two structural features: (i) considering the Baker-Nutting (B-N) orientation relationship between the NPs and the bcc grain^21^,^22^,^23^,^24^,^25^, NPs inside the planar arrays seem to grow predominantly towards the <100> _bcc_ direction. (ii) The linear NP chains assembling the NP arrays are aligned along the <110> _bcc_ direction as indicated by the dashed lines. To image one of the NPs along a different projection, a further magnified view of a representative NP marked by the purple box in Fig. 3a is shown in Fig. 3b. The chosen NP is elliptical in shape and its diameter along the<110> _bcc_ direction is 2.8 nm wide and 5.7 nm long. As determined by compositional profiles across the interfaces between the NP and the matrix, all of the NPs contain Mo (blue), V (green) and C (red).

**Figure 4:**
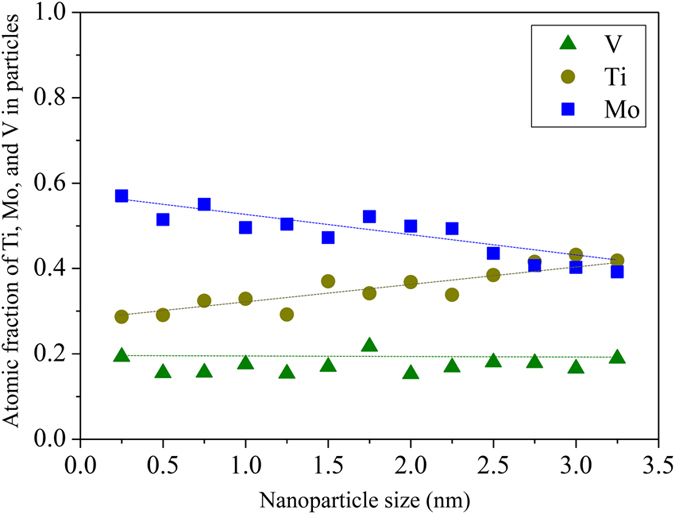
Chemical evolution of the nanoparticles found in all individual APT data sets of all annealed samples as a function of size. Atomic fraction of Ti, Mo, and V within all individual nanoparticles obtained from APT data sets for NP-30 and NP-60 samples by calculating the integral sum of Ti, Mo, and V.

Figure 4 shows the atomic fractions of Ti, Mo, and V inside the NPs for the heat treated samples as a function of NP size. The NPs have an average Ti:Mo:V compositional ratio of approx. 0.29:0.54:0.17 at the early nucleation stage, evolving towards 0.38 ± 0.04:0.44 ± 0.05:0.18 ± 0.02 at later growth stages. These results suggest that the early stages of NP nucleation are controlled mainly by atomic diffusion of Mo and C while subsequent NP growth is predominantly fed by diffusion of Ti and C. The V content of the NPs is size independent. Our observation of the transition from MoC-rich to TiMoC-rich NPs agrees with previous experimental findings^17^,^18^ and theoretical predictions^19^. For better understanding how the early-stage MoC rich NPs nucleate and how the Ti contributes to NP growth we performed APT characterization on larger NPs found on GBs in the NP-60 samples.

**Figure 5:**
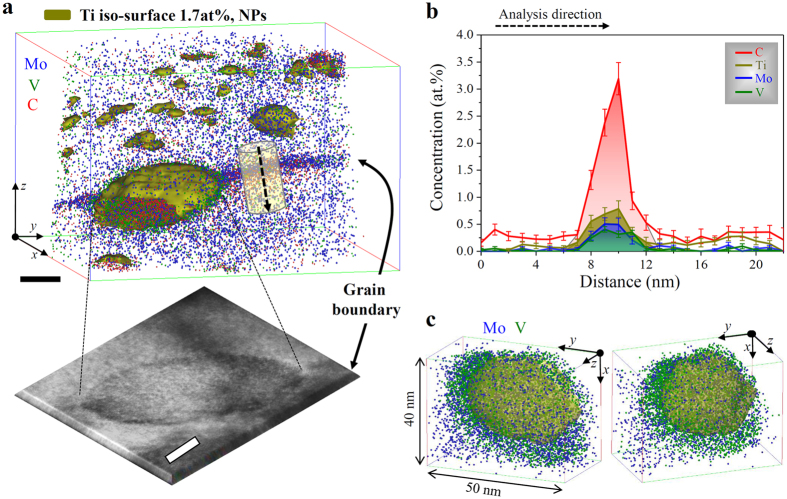
Coarsening of nanoparticles formed on a grain boundary in sample NP-60 (640 °C 60 min.). (**a**) 3D atom map of Mo (blue) and V (green) with a volume of 125 × 125 × 90 nm^3^ using 1.7 atomic % Ti isoconcentration surfaces (gold) revealing coarsening of the nanoparticle with a core/shell structure located on a grain boundary; scale bar, 20 nm. Blue: Mo, green: V, and red: C atoms. The tilted bright-field TEM micrograph, albeit not from the same exact NP, is shown to allow for visual correlation with the APT atom map. The TEM micrograph was tilted into the *x-y* plane to match the APT visualization of the nanoparticle formed on the grain boundary; scale bar, 5 nm. (**b**) Compositional profiles of particle-forming components across the matrix-particle interfaces along the dotted arrow highlighted in the yellow cylinder in (**a**). (**c**) Magnified 3D morphology of an elliptical core/shell structure of NP with two different projections in a volume of 40 × 50 × 30 nm^3^. Errors bars given in (**b**) represent the 2*σ* measurements.

Figure 5 shows an ultrahigh density of NPs as well as a single 15-nm elliptical NP formed at a GB alongside an illustrative electron micrograph obtained from a similar particle in the same material. The elemental concentrations along the cylindrical region marked in Fig. 5a are displayed in Fig. 5b (see compositional data at the GB or in the bulk phases in Supplementary Table S3). Assuming a dilute system, the Langmuir-Mclean isotherm allows for determination of the GB segregation coefficient *ß*_*i*_ of the diffusing solutes, which is also referred to as the GB enrichment factor, and is defined as the ratio between the proportion of a solute *i* at the boundary relative to its proportion in the bulk bcc grain. The *ß*_*i*_ values for C (6.4) and Mo (6.3) at the GBs exceed those of Ti (5.3) and V (4.8); i.e., Mo and C have stronger tendency to segregate to the GB compared to Ti and V. This result provides proof for the importance of the early-stage abundance of trace elements and their continuous redistribution during heat treatment to form MoC rich NPs.

Structural defects such as GBs or dislocations in materials are prone to solute segregation and have higher transport coefficients for diffusing atoms. Hence, such defects promote phase transformation when the temperature is high enough for diffusion^2^,^8^,^9^,^10^. Also, enhanced segregation and diffusion can accelerate coarsening of NPs at GBs or dislocations, thereby controlling their size and composition. At one such GB in a NP-60 sample, we observed coarsening of a NP, referred to as GB-NP, which has grown nearly five times larger than the non-coarsened NPs in the adjacent matrix (Fig. 5a,b). Two orthogonal views of the GB-NP (Fig. 5c) reveal a core-shell structure with a Ti-C rich core surrounded by a Mo-V-enriched shell. This finding reveals that the GBs promote a particle coarsening process by facilitating solute transport and segregation.

**Figure 6:**
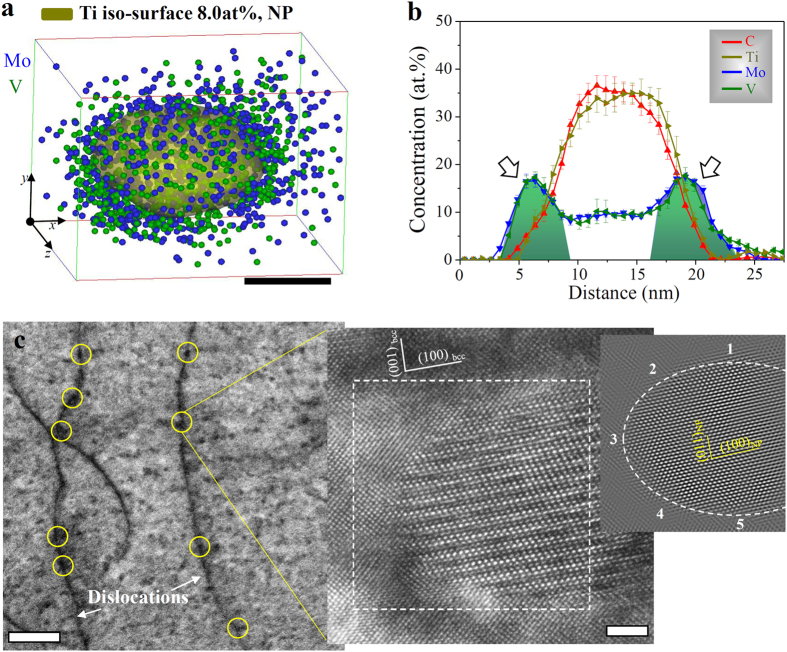
Coarsened nanoparticles formed on dislocations in sample NP-60 (640 °C 60 min.). (**a**) Isoconcentration surfaces of 8 atomic % Ti (yellow) exhibit a single core/shell nanoparticle in the 3D atom map with a volume of 18 × 15 × 18 nm^3^; scale bar, 5 nm. (**b**) Corresponding compositional profiles of particle-forming elements across the matrix-particle interfaces revealing a Ti-C rich core and a Mo-V-enriched shell structure (arrows). (**c**) Enlarged view of a bright-field TEM image taken from Fig. 2a showing a large number of coarsened nanoparticles on dislocations, as highlighted by yellow circles; scale bar, 50 nm and the corresponding HRTEM lattice image of the highlighted nanoparticle showing the crystal lattice of the particle and the adjacent bcc phase; scale bar, 2 nm. Also included is a HRTEM image, taken from the outlined area, after noise filtering by means of fast Fourier transformation and inverse fast Fourier transformation to estimate the lattice misfit strain between the particle and the adjacent bcc phase. Errors bars given in (**b**) represent the 2*σ* measurements.

Besides the GB-NPs, coarsening of NPs is also found on dislocations in both, the NP-60 (Fig. 6) and NP-360 samples (see Supplementary Fig. S2). The compositional profiles across the interfaces between the bcc matrix and the NP confirm that the NPs consist of a Ti-C rich core and a Mo-V-enriched shell. The APT-measured C content (36.2 ± 2.1 at. % as shown in Fig. 6b) in the particle core is depleted compared to an ideal stoichiometry of 50 at. % C of MC-type particles (M = Ti, Mo, V) with a perfect NaCl (B1) crystal structure. Because the introduction of C vacancies in the B1 crystal structure improves the stability of MC-type particles^28^,^29^, the observed C off-stoichiometry is attributed to vacancies in the Ti-C rich core. The Mo (9.1 ± 1.0 at. %) and V (9.3 ± 1.8 at. %) content in the particle core remain in solid solution. A large number of NPs in the NP-60 samples are coarsened on dislocations (Fig. 6c). To determine not only the crystal lattice parameter of NPs placed on dislocations but also their lattice misfit strain with the adjacent bcc phase, we performed HRTEM experiments. After filtering the noise from the lattice image by using fast Fourier transform (FFT) and inverse FFT analysis, the measured lattice parameter of the NP was determined as 0.445 nm, which is larger than that of TiC, (Ti,Mo)C or (Ti,V)C particles^19^,^23^,^24^. We observed that core/shell NPs located at dislocations exhibit a B1 crystal structure that obeys a B-N orientation relationship with the adjacent bcc grain^30^ (Fig. 3a and Supplementary Fig. S3).

## Discussion

As shown in Figs 1, 2, 3, a regularly architectured NP arrangement acts as an efficient obstacle array against dislocation motion. The contribution of regular particle arrays to the yield strength of the bcc Fe-based alloys had previously been studied^15^,^21^. Based on our results and those reported in literature, randomly dispersed obstacles are less efficient in strengthening as compared to regular precipitate arrays. Random particle fields contain dilute regions with larger spacings among the NPs. In such regions dislocations can bow out more easily and sweep large fractions of the slip plane since their bow-out stress scales inversely with the inter-NP spacing.

We also find that the NPs in the Ti–Mo–V doped and heat treated samples grow only weakly (Figs 2, 3, 4), i.e. the NPs resist capillary-driven coarsening. This is also reflected by the fact that the strength and ductility values remain practically unchanged over prolonged heating times.

Based on the current work and earlier findings^31^, it is plausible to conclude that the chemical potentials of Mo and C may be higher than those of Ti and V at the interfaces where the fcc → bcc solid phase transformation occurs at high temperatures, e.g. in the range of 800 °C. A difference in the elemental potentials may provide the difference in Gibbs free energy required for atomic diffusion, thereby promoting strong accumulation of Mo and C at the moving fcc/bcc interfaces during the fcc → bcc solid phase transformation. This phenomenon has two effects: (i) the higher compositional saturation of Mo and C than that of Ti and V leads to an increase in driving force for an early-stage nucleation of MoC-rich particles on the moving heterophase interfaces involved in the fcc → bcc transformation process at ~800 °C. (ii) As the temperature drops, the driving force increases with increasing nucleation rate, whereas particle growth slows down owing to the reduced diffusion rate. Therefore, in the final microstructure, the early-stage MoC rich NPs form chain-like planar arrays, which the moving interfaces leave behind during the fcc → bcc transformation process.

In the filtered HRTEM lattice image given in Fig. 6c, the lattice misfit strain (*δ*) between the core/shell particle and the bcc matrix was estimated according to ref. 32:


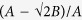


where, *A* is the measured lattice parameter of the NPs and *B* is the observed lattice parameter of the adjacent bcc matrix. The measured *δ* values reveal incoherent interfaces between the core/shell NPs and the bcc phase (see Supplementary Table S4). This observation indicates that despite the loss of coherency with the adjacent bcc grain caused by the formation of core/shell NPs, their orientation relationship was maintained.

In high-angle annular dark-field (HAADF) imaging using scanning TEM (STEM) the core regions of the dislocation-NPs show brighter contrast than the shell regions (see Supplementary Fig. S2c). This finding of a core-shell structured NPs in the NP-60 and NP-360 samples is consistent with the APT results (Figs 5c,6a and Supplementary Fig. S2). The combination of APT results and dark-field TEM conducted under appropriate diffraction conditions proves the existence of the core-shell structure for NPs located both, on GBs and dislocations. However, the NP shell did not produce any specific signal related to its crystalline structure in HRTEM: the interfacial accumulation of Mo and V at the particle shells represents an elemental enrichment phenomenon without any sign of structural transition or without producing any change in crystal structure. This suggests that the core/shell NPs grown on GBs or dislocations were formed via diffusion-controlled coarsening^33^,^34^.

Surprisingly, the core-shell NPs coarsened on GBs or dislocations in NP-60 samples (Figs 5c and 6a) are of similar size and composition as those found in the NP-360 samples (see Supplementary Fig. S2). This observation implies that the composition of the core-shell NPs remains practically constant, also upon prolonged heat treatment times. This means that capillarity–driven particle coarsening is impeded due to the interfacial segregation of Mo and V, which reduces the driving force for further particle coarsening due to a reduction in interface energy^10^. A schematic illustration of the entire transformation sequence summarizes the main evolution steps of the nanoparticles during cooling from the high-temperature fcc phase into the bcc phase (stages I and II), as shown in Supplementary Fig. S4. Also included are the final microstructures of the three different samples, depending on the nanoparticle size and composition. In samples NP-60 and NP-360, the coarsening of the Ti-C rich NPs on the GBs or dislocations was highlighted in terms of a gold core and a blue shell color coding.

In conclusion, we reported here about the formation of dense planar arrays of NPs which double the strength of a low-alloyed bcc steel. The NPs are of prevalent MoC and TiC composition at the early precipitation stages and assume a core-shell structure with a Ti-C rich core and a Mo-V rich shell after an incipient growth phase. The segregation of Mo and V to the particle/matrix interfaces yields size stabilization during heat treatment. These observations thus pave the pathway towards applying a simple bulk heat treatment for doubling the strength of steels via precipitation of highly regular core-shell NP arrays which resist coarsening, hence enabling alloy service in environments up to ~640 °C.

## Methods

### Sample preparation

The model alloy is a Fe–2.0 Mn–0.1 Si–0.1 Al–0.2 C steel containing a total of 0.3 at. % Ti, Mo, and V. The alloy was solution treated at 1250 °C for 240 min, then quenched to 920 °C, first hot-rolled to a thickness reduction of ~60% imposing a sequence of five subsequent rolling passes, and then further hot-rolled at 850 °C to a thickness reduction of ~80% using a sequence of six rolling passes to avoid further phase transformation during high-temperature deformation. The starting temperature of the fcc → bcc solid phase transformation was approx. 810 °C, and the transformation finishing temperature was 700 °C. The sample was quenched to 640 °C at a cooling rate of 25 °C/s, followed by isothermal heat treatments at 640 °C^15^,^18^,^24^ for 30, 60, and 360 min, respectively (Supplementary Fig. S1). The average grain size in the heat treated alloys was ~8.2 μm irrespective of the heat treatment times. For comparison, a Ti–Mo–V-free reference model material with the average grain size of ~10.5 μm was also isothermally heat treated at 640 °C for 60 min.

### Tensile testing

Tensile specimens with a gauge length of 12.5 mm and a diameter of 2.5 mm (ASTM E8 standard) were cut from a quarter through-thickness position for tensile testing. All samples used for tensile testing were prepared along the hot band transverse direction. The mechanical properties of all specimens in both, precipitate-free state and precipitation state were tested under uniaxial tension in an Instron Model 5582 test frame with a 100-kN load cell operated at a constant initial nominal strain rate of 1 × 10^−4^ s^−1^ at room temperature. The mechanical properties of all specimens were characterized in terms of ultimate tensile strength and 0.2% offset yield strength.

### Transmission electron microscopy

For TEM observations, 3 mm-diameter discs were punched out of the samples. Thin foils for TEM micrographs were prepared by electrolytic jet polishing. To observe the particles that formed on grain boundaries or at dislocations by HRTEM, samples were prepared by dual-beam focused ion beam (FIB, FEI Helios Nano-Lab^TM^). Bright-field low-magnified images, selected area diffraction patterns (SADPs), and HRTEM lattice images were obtained with a JEOL 2010F analytical TEM equipped with an aberration corrector which was operated at an acceleration voltage of 200 kV. Since uniform NP arrangements can be invisible under certain conditions by TEM imaging^22^, the [001]_bcc_ and [111]_bcc_ zone axis were chosen to display the arrangements of NPs and to identify the orientation relationship between the bcc matrix and the NPs. The carbon replica technique was employed on the heat treated samples to determine the number density of NPs per unit volume, with known inaccuracies for very small particles^22^. To measure the lattice parameters and the lattice mismatch between the NPs and the bcc host phase, we used fast Fourier transformation (FFT) to filter the noise from the lattice image, then performed inverse (IFFT) characterization of the targeted area. When performing FFT analysis, each diffraction pattern shown in the FFT diffractogram was identified by a Baker-Nutting orientation relationship.

### Local electrode APT

Samples for APT investigations were prepared by electro-polishing followed by a treatment with a dual-beam focused ion beam (FIB) (FEI, Helios Nano-Lab 600). APT analyses were conducted using a local electrode atom probe (LEAP 4000X HR, CAMECA^TM^) with a reflectron system in voltage-pulsing mode. The experimental parameters were set to maintain a 0.2% detection rate, 20% pulse fraction and 200-kHz pulse repetition. All measurements were performed at 60 K at <10^−7^ Pa pressure. At least, two or even more successful measurements were performed and evaluated, two of which contained more than 10 million ions. The APT data were visualized using the IVAS software (version 3.6.10) by Cameca Instruments. Reconstruction of APT maps was calibrated by determining plane spacing of bcc-crystal structure, based on detector event histograms observed in APT data sets^27^,^35^. The concentrations of C, Ti, and Mo were measured using the peak decomposition algorithm of IVAS^36^. Using the maximum separation algorithm, the NPs observed here were separated from the matrix, and the average composition of the NPs was calculated. This method can characterize microstructural features ranging from clusters to larger particles and secondary crystal phases by use of atomic-scale definitions including inter-atomic distances, local densities, and local atomic concentrations^27^,^36^. Statistical errors for measured atom counts were calculated as *σ = *(*C*_*i*_ × (*1* − *C*_*i*_)*/N*)^−1/2^, where *C*_*i*_ corresponds to measured atomic concentration fraction of the individual element *i* and *N* is the total atoms collected in the bin.

### Data availability

The data that support the findings of this study are available from the corresponding author upon request.

## Additional Information

**How to cite this article**: Seol, J.-B. *et al*. Core-shell nanoparticle arrays double the strength of steel. *Sci. Rep.* **7**, 42547; doi: 10.1038/srep42547 (2017).

**Publisher's note:** Springer Nature remains neutral with regard to jurisdictional claims in published maps and institutional affiliations.

## Supplementary Material

Supplementary Information

## References

[b1] Li Z., Pradeep K. G., Deng Y., Raabe D. & Tasan C. C. Metastable high-entropy dual-phase alloys overcome the strength–ductility trade-off. Nature 534, 227–230 (2016). 27279217 10.1038/nature17981

[b2] Kuzmina M. . Linear complexions: Confined chemical and structural states at dislocations. Science 349, 1080–1083 (2015). 26339026 10.1126/science.aab2633

[b3] Valiev R. Nanostructuring of metals by severe plastic deformation for advanced properties. Nat. Mater. 3, 511–516 (2004). 15286754 10.1038/nmat1180

[b4] Wang Y. . High tensile ductility in a nanostructured metal. Nature 419, 912–915 (2002). 12410306 10.1038/nature01133

[b5] Khalajhedayati A. . Manipulating the interfacial structure of nanomaterials to achieve a unique combination of strength and ductility. Nat. Commun. 7, 10802 (2016). 26887444 10.1038/ncomms10802 PMC4759628

[b6] Chen G. . Polysynthetic twinned TiAl single crystals for high-temperature applications. Nat. Mater. 15, 876–881 (2016). 27322822 10.1038/nmat4677

[b7] Herbig M. . Atomic-Scale Quantification of Grain Boundary Segregation in Nanocrystalline Material. Phys. Rev. Lett. 112, 126103 (2014). 24724663 10.1103/PhysRevLett.112.126103

[b8] Kirchheim R. Reducing grain boundary, dislocation line and vacancy formation energies by solute segregation. I. Theoretical background. Acta Mater. 55, 5129–5138 (2007).

[b9] Raabe D. . Segregation engineering enables nanoscale martensite to austenite phase transformation at grain boundaries: A pathway to ductile martensite. Acta Mater. 61, 6132–6152 (2013).

[b10] Raabe D. . Grain boundary segregation engineering in metallic alloys: A pathway to the design of interfaces. Curr. Opin. Solid State Mater. Sci. 18, 253–261 (2014).

[b11] Vogel F. . Mapping the evolution of hierarchical microstructures in a Ni–based superalloy. Nat. Commun. 4, 2955 (2013). 24356413 10.1038/ncomms3955

[b12] Devaraj A. . A low-cost hierarchical nanostructured beta-titanium alloy with high strength. Nat. Commun. 7, 11176 (2016). 27034109 10.1038/ncomms11176 PMC4821990

[b13] Orowan E. In Precipitation hardening (Oxford, Pergamon Press, 1968).

[b14] Ashby M. F. In Oxide dispersion strengthening (New York, Gordon and Breach, 1958).

[b15] Huang Y. . A high-strength high-ductility Ti- and Mo-bearing ferritic steel. Metall. Mater. Trans. A 47, 450–460 (2016).

[b16] Honeycombe R. W. K. & Mehl R. F. Transformation from austenite in alloy steels. Metall. Trans. 7A, 915–936 (1976).

[b17] Ricks R. A. & Howell P. R. The formation of discrete precipitate dispersions on mobile interphase boundaries in iron-base alloys, Acta Metall. 31, 853–861 (1983).

[b18] Funakawa Y. . Development of high strength hot-rolled sheet steel consisting of ferrite and nanometer-sized carbides. ISIJ Int. 44, 1945–1951 (2004).

[b19] Jang J. H., Lee C. H., Heo Y. U. & Suh D. W. Stability of (Ti, M)C (M = Nb, V, Mo and W) carbide in steels using first–principles calculations. Acta Mat. 60, 208–217 (2012).

[b20] Wei F. G., Hara T. & Tsuzaki K. High resolution transmission electron microscopy study of crystallography and morphology of TiC precipitates in tempered steel. Phil. Mag. 11, 1735–1751 (2001).

[b21] Yen H. W., Chen P. Y., Huang C. Y. & Yang J. R. Interphase precipitation of nanometer-sized carbides in a titanium-molybdenum–bearing low-carbon steel. Acta Mater. 59, 6264–6274 (2011).

[b22] Yen H. W., Huang C. Y. & Yang J. R. Characterization of interphase-precipitated nanometer-sized carbides in a Ti–Mo–bearing steel. Scr. Mater. 61, 616–619 (2009).

[b23] Timokhina I. B. . Precipitate characterization of an advanced high-strength low-alloy (HSLA) steel using atom probe tomography. Scr. Mater. 56, 601–604 (2007).

[b24] Mukherjee S. . Three–dimensional atom probe microscopy study of interphase precipitation and nanoclusters in thermomechanically treated titanium–molybdenum steels. Acta Mat. 61, 2521–2530 (2013).

[b25] Yen H. W., Huang C. Y. & Yang J. R. The nano carbide control: design of super ferrite in steels. Adv. Mater. Res. 89–91, 663–668 (2010).

[b26] Vetter T., Iggland M., Ochsenbein D. R., Hänseler F. S. & Mazzotti M. Modeling Nucleation, Growth, and Ostwald Ripening in Crystallization Processes: A Comparison between Population Balance and Kinetic Rate Equation. Cryst. Growth Des. 13, 4890–4905 (2013).

[b27] Gault B., Moody M. P., Cairney J. M. & Ringer S. P. Atom Probe Microscopy (Springer Science+Business Media, 2012).

[b28] Hugosson H. W., Jansson U., Johansson B. & Eriksson O. Phase stability diagrams of transition metal carbides, a theoretical study. Chem. Phys. Lett. 333, 444–450 (2001).

[b29] Lipatnikov V. N. . Effects of vacancy ordering on structure and properties of vanadium carbide. J. Alloys Compd. 261, 192–197 (1997).

[b30] Baker R. G. & Nutting J. Precipitation processes in steels, Special Report **64** (London: Iron and Steel Institute) 1–22 (1959).

[b31] Gouné M. . Overview of the current issues in austenite to ferrite transformation and the role of migrating interfaces therein for low alloyed steels. Mater. Sci. Eng. R. 92, 1–38 (2015).

[b32] Charleux M., Poole W. J., Militzer M. & Deschamps A. Precipitation behavior and its effect on strengthening of an HSLA-Nb/Ti steel. Metall. Mater. Trans. A 32, 1635–47 (2001).

[b33] Ardell A. J. & Ozolins V. Trans–interface diffusion–controlled coarsening. Nat. Mater. 4, 309–316 (2005). 15778716 10.1038/nmat1340

[b34] Kapoor M., O’malley R. & Thompson G. B. Atom probe tomography study of multi-miscoalloyed carbide and carbo-nitiride precipitates and the precipitation sequence in Nb-Ti HSLA steels. Metall. Mater. Trans. A 47, 1984–95 (2016).

[b35] Gault B. . Advances in the reconstruction of atom probe tomography data. Ultramicroscopy 111, 448–457 (2011). 21146931 10.1016/j.ultramic.2010.11.016

[b36] Larson D. J. . Local Electrode Atom Probe Tomography: A User’s Guide (Springer, 2013).

