# Supplementary material for: Core-shell nanoparticle arrays double the strength of steel
Source: Sci Rep. 2017 Feb 22;7:42547. doi: 10.1038/srep42547 (PMC5320495; doi:10.1038/srep42547)
Supplement: Supplementary Information [file srep42547-s1.pdf]

SUPPLEMENTARY INFORMATION

Core-shell nanoparticle arrays double the strength of steel

J.-B. Seol[1†], S.-H. Na[2†], B. Gault[3†], J.-E. Kim[2], J.-C. Han[2], C.-G. Park[1,2] & D. Raabe[3]**

Supplementary Figure S1. Schematic illustration of thermal heat treatments of the model alloys containing Ti, Mo and V, and of the Ti-Mo-V free reference sample. The material is an Fe–2.0 Mn–0.2 Si–0.1 Al–0.2 C alloy containing a total of 0.3 at. % Ti, Mo, and V. Samples subjected to heat treatments at 640 ℃ for 30, 60, or 360 min are referred to as NP-30, NP-60, and NP-360, respectively.

Supplementary Figure S2. (**a**) Two different projections of 3D atom maps of a coarsened nanoparticle in sample NP-360 (640 ℃ 360min.), using 8.0 atomic % Ti isoconcentration surfaces (gold) showing the magnified 3D morphology of an elliptical core/shell structure of NP; scale bar, 5 nm. (**b**) Compositional profiles of particle-forming components across the matrix-particle interfaces. (**c**) Representative high-angle annular dark-field imaging using a scanning TEM showing a difference in the contrast between core and shell regions of two nanoparticles; scale bar, 20 nm. Errors bars given represent the 2σ measurements.

Supplementary Figure S3. Fast Fourier transformation diffractogram taken from the rectangular region in HRTEM image (figure 6c) and a corresponding schematic illustration revealing that the nanoparticle matches a Baker-Nutting orientation relationship with the adjacent bcc phase (lines and circles). Red line and circles: bcc crystal phase, yellow lines and circles: particle spots. Extra diffraction spots (black circles) may originate from the formation of iron-based oxides on the surface of TEM specimens, and to the double diffraction from oxide and bcc[22].

Supplementary Figure S4. Schematic diagram showing the main evolution steps of the nanoparticles during cooling from the high-temperature fcc phase into the bcc phase (stages I and II), and the corresponding final microstructures of samples NP-30 (640 ℃ 30min.), NP-60 (640 ℃ 60min.) and NP-360 (640 ℃ 360min.). Green and gold colored particles indicate the early-stage Mo-C rich NPs with a size of <0.5 nm and non-coarsened Ti-C rich NPs with a size of <3.5 nm.

Supplementary Table S1. Comparison of the mechanical properties of the present NP-free and NP-containing alloys with similar materials, doped with Ti and Mo, presented before in the literature[15,18]. UTS, ultimate tensile strength; YS, yield strength (at 0.2% offset); EL, elongation (strain to fracture).

Supplementary Table S2. Average number density of nanoparticles observed in the heat treated samples. APT, atom probe tomography; TEM, transmission electron microscopy; ND, not detected.

Supplementary Table S3. Chemical analysis of an isothermally treated alloy. APT, atom probe tomography; ND, not detected.

Supplementary Table S4. Quantities determined by HR-TEM; Lattice parameters and interface

misfit strain around and within nanostructures. HR-TEM, high-resolution transmission electron microscopy.

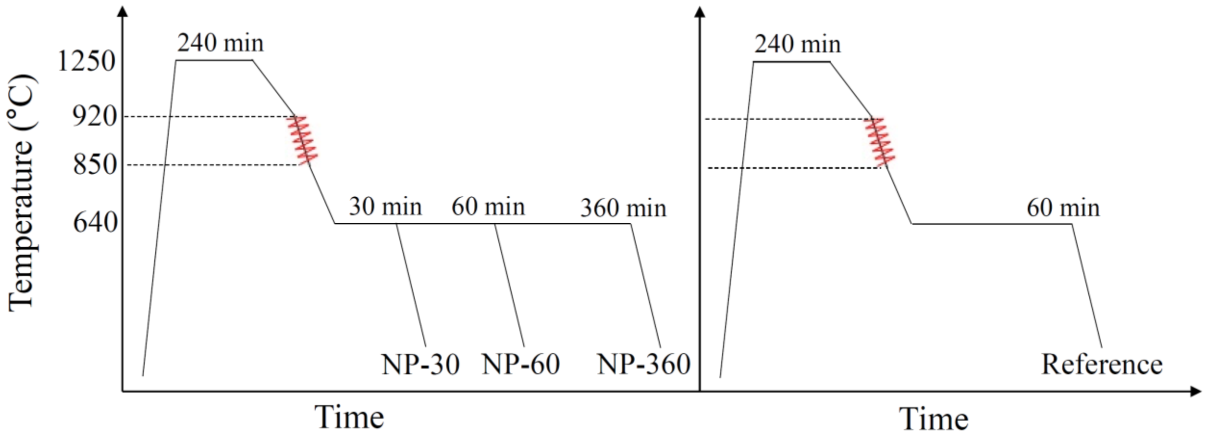

Supplementary Figure S1. Schematic illustration of thermal heat treatments of the model alloys containing Ti, Mo and V, and of the Ti-Mo-V free reference sample. The material is an Fe–2.0 Mn–0.2 Si–0.1 Al–0.2 C alloy containing a total of 0.3 at. % Ti, Mo, and V. Samples subjected to heat treatments at 640 °C for 30, 60, or 360 min are referred to as NP-30, NP-60, and NP-360, respectively.

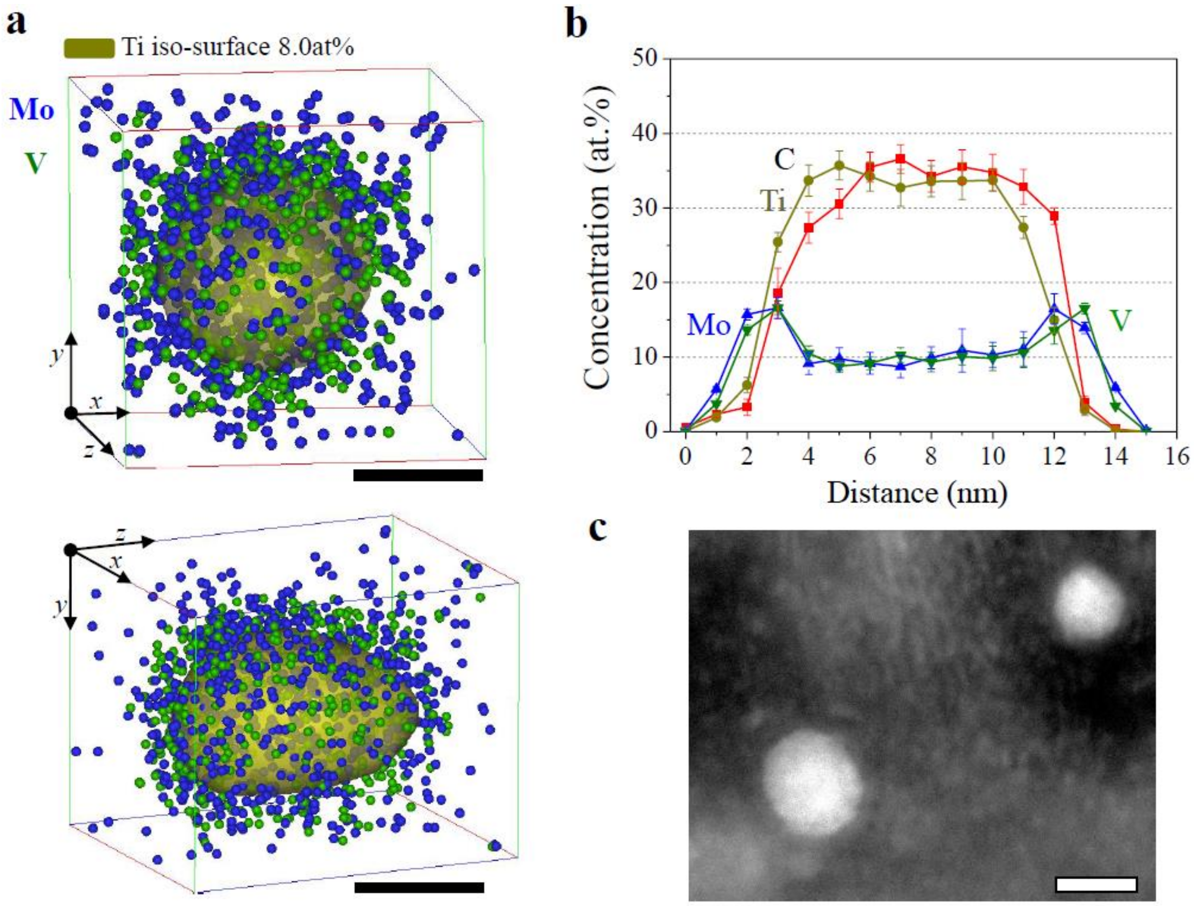

Supplementary Figure S2. (**a**) Two different projections of 3D atom maps of a coarsened nanoparticle in sample NP-360 (640 °C 360min.), using 8.0 atomic % Ti isoconcentration surfaces (gold) showing the magnified 3D morphology of an elliptical core/shell structure of NP; scale bar, 5 nm. (**b**) Compositional profiles of particle-forming components across the matrix-particle interfaces. (**c**) Representative high-angle annular dark-field imaging using a scanning TEM showing a difference in the contrast between core and shell regions of two nanoparticles; scale bar, 20 nm. Errors bars given represent the 2σ measurements.

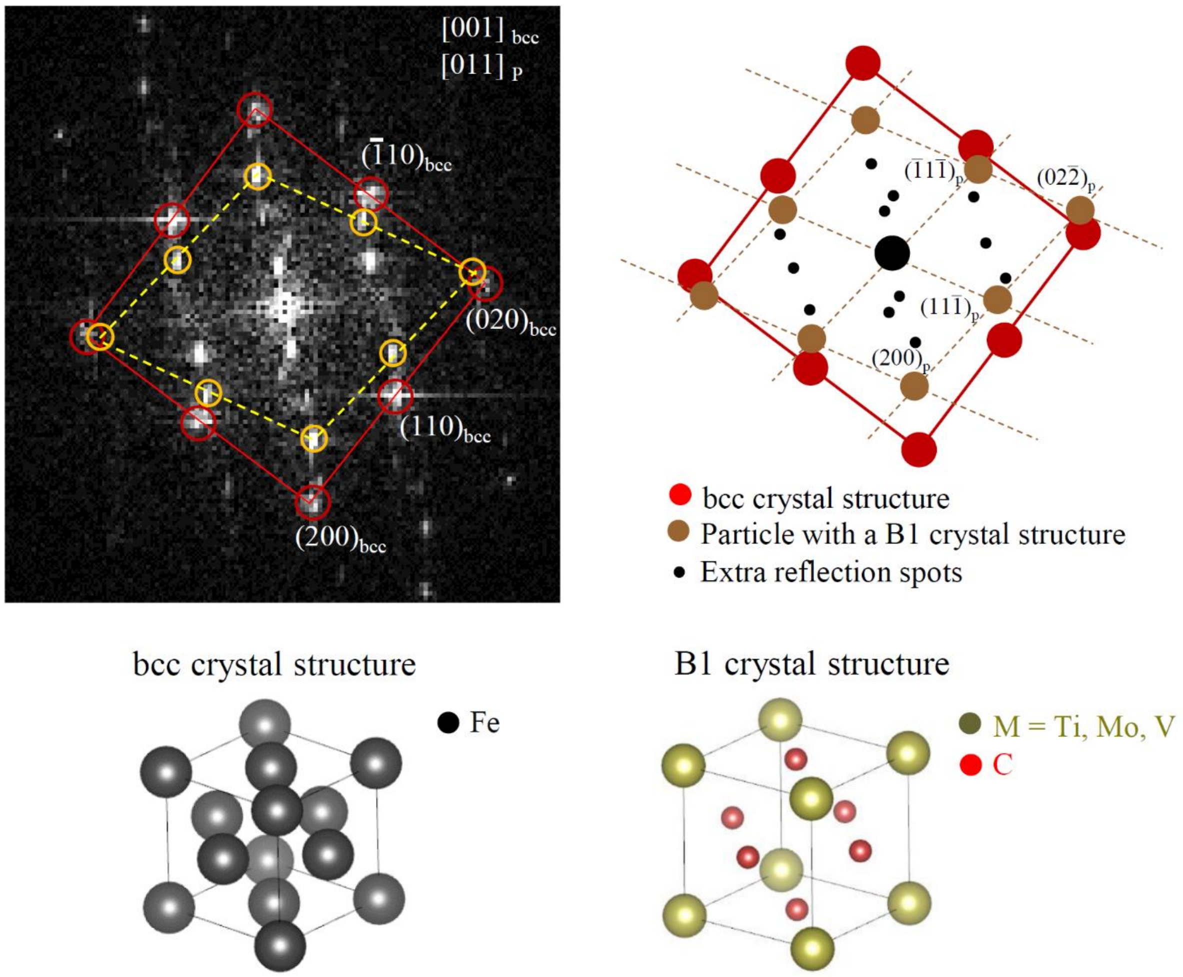

Supplementary Figure S3. Fast Fourier transformation diffractogram taken from the rectangular region in HRTEM image (figure 5c) and a corresponding schematic illustration revealing that the nanoparticle matches a Baker-Nutting orientation relationship with the adjacent bcc phase (lines and circles). Red line and circles: bcc crystal phase, yellow lines and circles: particle spots. Extra diffraction spots (black circles) may originate from the formation of iron-based oxides on the surface of TEM specimens, and to the double diffraction from oxide and bcc[22].

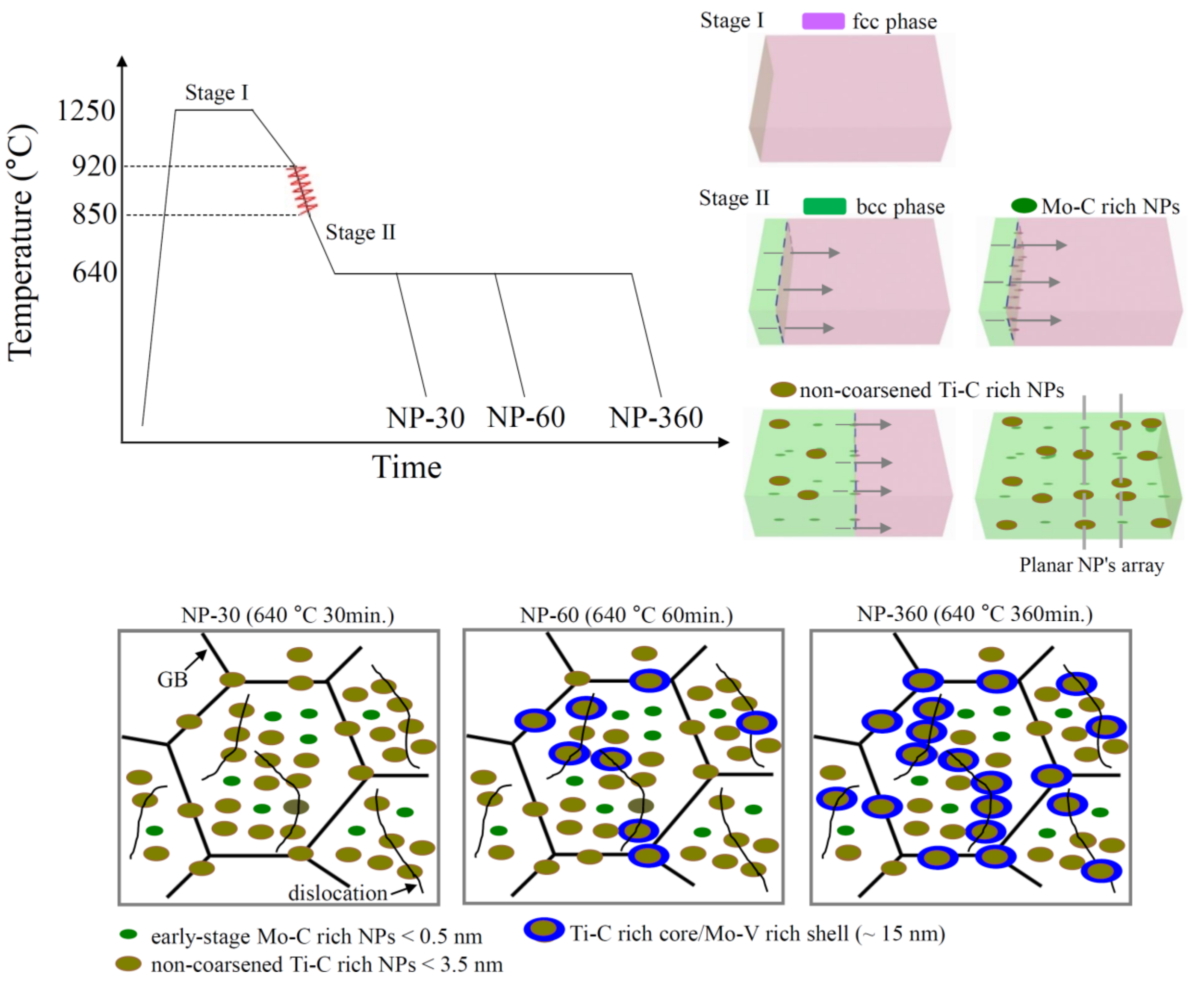

Supplementary Figure S4. Schematic diagram showing the link between the employed heat treatment and the phase transformation. Green and gold colored particles indicate the early-stage Mo-C rich NPs with a size of <0.5 nm and un-coarsened Ti-C rich NPs with a size of <3.5 nm.

Supplementary Table S1. Comparison of the mechanical properties of the present NP-free and NP-containing alloys with similar materials, doped with Ti and Mo, presented before in the literature[15,18]. UTS, ultimate tensile strength; YS, yield strength (at 0.2% offset); EL, elongation (strain to fracture).

	**Composition (wt.%)**	**Heat treatment, temperature (°C) / time**	**YS (MPa)**	**UTS (MPa)**	**EL (Pct)**
**Huang Y. et al.**	Fe-0.09C-0.21Si-1.71Mn-0.37Mo-0.19Ti	700 / 60 min.	670	720	24.64
		625 / 60 min.	835	870	21.48
		600 / 60 min.	945	995	20.18
**Funakawa Y. et al**	Fe-0.047C-0.22Si-1.59Mn-0.2Mo-0.82Ti	648 / 60 min.	734	807	24
**This study**	Ti-Mo-V-doped sample	640 / 30 min.	810 ± 38	967 ± 38	18.66
		640 / 60 min.	840 ± 24	1000 ± 20	19.97
		640 / 360 min.	837 ± 30	980 ± 15	18.91
	Un-doped reference sample	640 / 60 min.	400 ± 19	500 ± 25	17.08

Supplementary Table S2. Average number density of nanoparticles observed in the heat treated samples.

Samples	Average nanoparticle number density	
	Measured by APT	Measured by TEM
**Reference**	ND	ND
**NP–30**	$2.2 \times 10^{23}\ m^{-3}$	$1.6 \times 10^{22}\ m^{-3}$
**NP–60**	$6.8 \times 10^{23}\ m^{-3}$	$2.5 \times 10^{22}\ m^{-3}$
**NP–360**	$4.6 \times 10^{23}\ m^{-3}$	$2.7 \times 10^{22}\ m^{-3}$

APT, atom probe tomography; TEM, transmission electron microscopy; ND, not detected.

Supplementary Table S3. Chemical analysis of an isothermally treated alloy.

	Measured composition of all solute elements at the grain boundary (GB) or inside bulk phases (at.%)			Nominal composition of an alloy (at.%)
	GB	Grain #1	Grain #2	
**Fe**	91.87±0.49	98.40±0.50	98.43±0.53	97.16
**Mn**	3.10±0.25	1.15±0.25	1.22±0.20	2.01
**Si**	0.18±0.04	0.09±0.03	0.11±0.03	0.16
**C**	3.19±0.31	0.06±0.02	0.05±0.02	0.25
**Al**	0.07±0.02	0.08±0.03	0.07±0.02	0.07
**Ti**	0.78±0.23	0.18±0.06	0.25±0.06	0.11
**Mo**	0.49±0.22	ND	ND	0.12
**V**	0.32±0.06	0.02±0.01	0.02±0.01	0.12

APT, atom probe tomography; ND, not detected.

Supplementary Table S4. Quantities determined by HR-TEM; Lattice parameters and interface misfit strain around and within nanostructures.

	Lattice parameter, nm	Lattice misfit between matrix and individual regions
bcc matrix	0.289	-
Region 1	0.434	4.61 %
Region 2	0.457	9.43 %
Region 3	0.428	3.27 %
Region 4	0.473	9.51 %
Region 5	0.421	2.13 %
Particle center	0.445	6.97 %

HR-TEM, high-resolution transmission electron microscopy.
